# Effect of Seasonal Variation on Adult Clinical Laboratory Parameters in Rwanda, Zambia, and Uganda: Implications for HIV Biomedical Prevention Trials

**DOI:** 10.1371/journal.pone.0105089

**Published:** 2014-08-13

**Authors:** Eugene Ruzagira, Andrew Abaasa, Etienne Karita, Joseph Mulenga, William Kilembe, Susan Allen, Ubaldo Bahemuka, Agnes N. Bwanika, Jonathan Levin, Matthew A. Price, Anatoli Kamali

**Affiliations:** 1 Medical Research Council (MRC)/Uganda Virus Research Institute (UVRI) Uganda Research Unit on AIDS, Entebbe, Uganda; 2 Projet San Francisco (PSF), Kigali, Rwanda; 3 Zambia-Emory HIV Research Project (ZEHRP), Lusaka, Zambia; 4 Zambia Blood Transfusion Service, Lusaka, Zambia; 5 Department of Global Health, Rollins School of Public Health, Emory University, Atlanta, Georgia, United States of America; 6 International AIDS Vaccine Initiative, New York, New York, United States of America; 7 Department of Epidemiology and Biostatistics, University of California at San Francisco, San Francisco, California, United States of America; Johns Hopkins Bloomberg School of Public Health, United States of America

## Abstract

**Objectives:**

To investigate the effect of seasonal variation on adult clinical laboratory parameters in Rwanda, Zambia, and Uganda and determine its implications for HIV prevention and other clinical trials.

**Methods:**

Volunteers in a cross-sectional study to establish laboratory reference intervals were asked to return for a seasonal visit after the local season had changed from dry to rainy or vice versa. Volunteers had to be clinically healthy, not pregnant and negative for HIV, Hepatitis B and C, and syphilis infection at both visits. At each visit, blood was taken for measurement of hemoglobin, haematocrit, mean corpuscular volume, red blood cells, platelets, total white blood cells (WBC), neutrophils, lymphocytes, monocytes, eosinophils, basophils, CD4/CD8 T cells, aspartate aminotransferase, alanine aminotransferase, alkaline phosphatase, direct bilirubin, total bilirubin, total immunoglobulin gamma, total protein, creatinine, total amylase, creatine phosphokinase and lactate dehydrogenase (LDH). Consensus dry season reference intervals were applied to rainy season values (and vice versa) and the proportion of ‘out-of-range’ values determined. Percentage differences between dry and rainy season parameter mean values were estimated.

**Results:**

In this cohort of 903 volunteers, less than 10.0% of consensus parameter (except LDH) values in one season were “out-of-range” in the other. Twenty-two (22) percent of rainy season LDH values fell outside of the consensus dry season interval with the higher values observed in the rainy season. Variability between consensus seasonal means ranged from 0.0% (total WBC, neutrophils, monocytes, basophils, and direct bilirubin) to 40.0% (eosinophils). Within sites, the largest seasonal variations were observed for monocytes (Masaka, 11.5%), LDH (Lusaka, 21.7%), and basophils (Kigali, 22.2%).

**Conclusions:**

Seasonality had minimal impact on adult clinical laboratory parameter values in Rwanda, Zambia, and Uganda. Seasonal variation may not be an important factor in the evaluation of adult clinical laboratory parameters in HIV prevention and other clinical trials in these countries.

## Introduction

Reference intervals of clinical laboratory values may be influenced by endemic pathogens [1]–[3], nutritional [1], [4], genetic [5]–[7], physiologic [8], environmental [2], [9], and/or socioeconomic [10] factors. In response to the growing number of clinical trials in sub-Saharan Africa, several studies have been conducted to establish locally relevant clinical laboratory reference values [2], [3], [11]–[15]. However, only a few studies have evaluated the effect of seasonal variation (rainy versus dry season) on clinical laboratory parameters in African populations. In a study among HIV negative pregnant women in Zimbabwe, neutrophil and lymphocyte counts were higher in the rainy compared to the dry season while monocyte counts were higher in the dry compared to the rainy season [4]. In the same population, CD4 T cell counts were higher in the rainy compared to the dry season [16]. Among healthy West African children, rainy season CD4 T cell counts were significantly lower than those obtained in the dry season whereas CD8 T cell percent was higher during the rainy season compared to the dry season [17]. These findings suggest that seasonal variation may influence certain clinical laboratory parameter values. This raises the question of whether season should be taken into account during evaluation of clinical laboratory parameters in clinical trials conducted in sub-Saharan Africa.

In the current study, we assessed the effect of seasonal variation on hematological and biochemistry parameters among potential volunteers for HIV biomedical prevention clinical trials in Rwanda, Zambia, and Uganda in order to establish whether season may be an important factor in the evaluation of adult clinical laboratory parameters in HIV prevention and other clinical trials in these countries.

## Methods

### Ethical statement

The study was approved by the National Ethics Committee of Rwanda, the University of Zambia Biomedical Research Ethics Committee, the Emory University School of Public Health Ethics Committee, the Uganda Virus Research Institute Science and Ethics Committee, and the Uganda National Council for Science and Technology. Written informed consent was obtained from each volunteer before study procedures were conducted.

### Study population

The study was nested in the International AIDS Vaccine Initiative (IAVI) African laboratory reference intervals study whose aim was to establish laboratory reference intervals in clinically healthy adult (18–60 years) Africans [3]. Study participants were recruited at three clinical research centres in Kigali, Rwanda; Lusaka, Zambia; and Masaka, Uganda. The methods used in the IAVI African laboratory reference intervals study have been described previously, including details on the source population and screen outs [18]. Between 200 and 400 volunteers (50% women by design) were recruited at each research centre, as recommended by CLSI guidelines for characterizing laboratory reference intervals (CLSI, www.clsi.org) [18].

#### Enrolment (Visit 1)

At enrolment into the IAVI African laboratory reference intervals study, detailed demographic data and comprehensive medical history were obtained. A full general physical examination including measurements of vital signs (blood pressure, pulse rate, respiratory rate, and temperature) was performed. Blood and urine samples were collected. HIV counselling and testing was performed if the volunteer did not have a documented negative HIV test result performed in the previous four weeks. A follow-up visit was conducted 2–4 weeks after enrolment to provide volunteers with laboratory test results from the enrolment visit. Volunteers who had abnormal results were asked to provide fresh samples for repeat testing. Those that were confirmed to have clinically significant laboratory test values and/or medical conditions were given appropriate clinical care and referred for further evaluation and treatment as necessary. Volunteers were excluded from analysis if they had clinically significant history and/or examination findings, or if laboratory tests showed that they were pregnant, positive for HIV-1/2, Hepatitis B surface antigen (HBsAg), antibodies against hepatitis C or RPR (suspected syphilis).

#### Seasonal visit (Visit 2)

Volunteers that were eligible for analysis in the IAVI African laboratory reference intervals study were scheduled to to attend a second (seasonal) visit at least six months after enrolment but within the first year. Volunteers that had been enrolled in the rainy season were scheduled to return in the next dry season and vice versa. At the seasonal visit, an interim medical history was obtained, a symptom-directed physical exam including measurements of vital signs was performed, and all laboratory tests performed at visit 1 repeated. A follow-up visit was conducted 2–4 weeks later to provide volunteers with results, repeat tests in case of abnormal results, provide clinical care and referrals. Similar criteria to those described for visit 1 were applied to exclude volunteers from analysis at visit 2.

#### Analyzable population

The analyzable population for the current study comprised of only volunteers that were analysed at visit 1 and that were also eligible for inclusion in analysis at visit 2.

#### Determination of season

The study was conducted between December 2004 and September 2006. In general, the rainy season occurs from October to April in Zambia [19], February to July, and October to January in Rwanda [20], March to May, and October to December in Uganda [21], [22]. There is however considerable regional and inter-annual seasonal variation [19], [21]. Therefore site investigators assigned season (rainy or dry) based on best judgment at enrolment (visit 1). Seasonal visits (visit 2) were pre-determined based on the estimated timing of seasonal change. Rain gauge data were collected during the study. At the end of study, these data were summarized and used to confirm the seasonal assignments.

At Lusaka, study visits were conducted from November 2005 to March 2006 (rainy season), and May to September 2006 (dry season). The site’s average monthly rainfall was 166.6 mm and 0.0 mm for the rainy and dry seasons respectively. At Kigali, study visits were conducted from December 2004 to May 2005 (rainy season) and June to August 2005 (dry season). It was not possible to retrieve rain gauge data for this site. At Masaka, rainy season visits were conducted from October to November 2005 (average monthly rainfall, 95.5 mm) and March to May 2006 (average monthly rainfall, 163.3 mm). Dry season visits were conducted from June to September 2005 (average monthly rainfall, 29.8 mm) and December 2005 to February 2006 (average monthly rainfall, 38.6 mm).

### Laboratory evaluation

The laboratory methods used in this study have been previously described [3], [18]. All participating laboratories were enrolled in a quality assurance/quality control program with Clinical Laboratory Services (CLS), Johannesburg, South Africa to assure accuracy and comparability of all test results across time and research centers. Blood was screened for HIV-1/2, Hepatitis B, Hepatitis C, and Syphilis. Urine was screened for hCG (females), protein, blood, glucose, ketones, esterase, and nitrite. Urine microscopy was performed for volunteers who had abnormalities of ≥2+ protein or blood or positive leucocyte esterase or nitrite on dipstick analysis.

The following haematology, immunology, and biochemistry parameters were evaluated: hemoglobin, haematocrit, mean corpuscular volume, red blood cells (RBC), platelet, total white blood cell (WBC), neutrophil, lymphocyte, monocyte, eosinophil, and basophil counts; CD4 and CD8 counts; aspartate aminotransferase (AST), alanine aminotransferase (ALT), alkaline phosphatase (ALP), direct bilirubin, total bilirubin, total immunoglobulin gamma, total protein, creatinine, total amylase, creatine phosphokinase (CPK) and lactate dehydrogenase (LDH).

Samples for CD4 and CD8 T cell count determination were obtained before noon to minimize the effect of CD4 diurnal variation.

### Statistical methods

Data were recorded on source documents and transcribed on Case Report Forms which were then faxed to a central server using DataFax software (Clinical DataFax Systems Inc., Hamilton, Canada). All data analyses were conducted using Stata 11.0 (College Park, TX, USA). The Clinical and Laboratory Standards Institute (CLSI, www.clsi.org) terms and guidelines for defining reference intervals were followed. Baseline characteristics were presented using counts and percentages. We used the following criteria to obtain consensus seasonal (rainy or dry) reference intervals. We compared the data across study sites and volunteer gender using the p-values obtained from the overall ANOVA, which were adjusted for multiple comparisons using the Tukey method. If not statistically significantly different, then the data were combined first across sites, then across volunteer gender. If significantly different, but the difference between means was less than 25% of the width of the 95% reference interval estimated from the combined dataset, and the ratio of standard deviations was less than 1.5, then the data from the two sites (or genders) were combined. For parameter data that were not normally distributed, all ANOVA tests were performed after a log transformation. Where data of a given site or gender violated the fore stated characteristics, the analyte’s consensus reference interval was estimated without that site’s or gender’s data. Consensus rainy and dry season laboratory reference intervals were estimated as the 2.5^th^ and 97.5^th^ percentiles of the combined datasets thereby including 95% of the data for a given analyte [23]. We estimated the proportions of rainy season values that fell outside the consensus dry season laboratory reference interval and vice-versa. We further estimated the consensus and site level rainy and dry season laboratory parameter means and medians (consensus only). We defined the magnitude of seasonal variation as the percentage difference between the rainy and dry season laboratory parameter mean values [difference between rainy and dry season laboratory parameter mean values/average of the rainy and dry season laboratory parameter mean values) ×100]. We did not use standard statistical methods to compare seasonal laboratory parameter values as our sample sizes were typically large enough to detect very small but clinically insignificant differences between seasons.

## Results

### Study population

A total of 1604 volunteers [Lusaka (497), Kigali (505), and Masaka (602)] were screened for the IAVI African laboratory reference intervals study. Of these, 1058 (66%) volunteers [Lusaka (352), Kigali (373), and Masaka (333)] were included in the laboratory reference intervals analysis (visit 1). Detailed reasons for screen out are presented in [18] and summarized in Table 1. Screened-out volunteers were older than those enrolled (Mean age: 32 versus 31 years, student’s t-test: p = 0.02). More women than men were screened-out but this difference was not statistically significant (35.9% versus 32.2%, chi-square test: p = 0.11).

**Table 1:** Summary of reasons for screen-outs, non-attendance of seasonal visit and exclusion from seasonal analysis by site.

	Lusaka		Kigali		Masaka		All sites	
	N	%	N	%	N	%	N	%
**Screened**	**497**		**505**		**602**		**1604**	
**Volunteers screened-out** #	**145**	**29.2**	**132**	**26.1**	**269**	**44.7**	**546**	**30.0**
Splenomegaly	1	0.2	7	1.4	78	12.9	86	5.4
Hypertension	22	4.4	6	1.2	22	3.7	50	3.1
Flu like symptoms	7	1.4	9	1.8	22	3.7	38	2.4
Sexually transmitted infection	9	1.8	4	0.8	21	3.5	34	2.1
Low body-mass index	7	1.4	13	2.6	11	1.8	31	1.9
Acute respiratory infections	3	0.6	8	1.6	7	1.2	18	1.1
HIV antibody positive	1	0.2	1	0.2	0	0.0	2	0.1
Other medical history/examination abnormality	39	7.8	32	6.3	45	7.5	116	7.2
Menstruation	18	3.6	13	2.6	4	0.7	35	2.2
Pregnant	2	0.4	0	0.0	11	1.8	13	0.8
Unable to provide informed consent	2	0.4	17	3.4	27	4.5	46	2.9
Other non-medical reasons	8	1.6	8	1.6	8	1.3	24	1.5
Hepatitis B antigen positive	23	4.6	13	2.6	5	0.8	41	2.6
Hepatitis C antibody positive	6	1.2	10	1.9	37	6.1	53	3.3
Syphilis/RPR positive	11	2.2	0	0.0	21	3.5	32	1.9
Pregnant	1	0.2	4	0.8	2	0.3	7	0.4
Other	2	0.4	0	0.0	11	1.8	13	0.8
**Analyzed at visit 1**	**352**		**373**		**333**		**1058**	
**Missed seasonal visit (visit 2)**	**53**	**15.1**	**10**	**2.7**	**45**	**13.5**	**108**	**10.2**
Lost to follow-up	50	14.2	9	2.4	43	12.9	102	9.6
Withdrawn by investigator due to non-compliance	1	0.3	0	0.0	1	0.3	2	0.2
Volunteer requested to discontinue	2	0.6	1	0.0	1	0.3	4	0.4
**Attended seasonal visit (visit 2)**	**299**		**363**		**288**		**950**	
**Excluded from seasonal visit analysis**	**10**	**3.3**	**17**	**4.7**	**20**	**6.9**	**47**	**4.9**
Clinically significant abnormality on physical examination	0	0.0	4	1.1	0	0.0	4	0.4
Hospitalization in last 6 months	0	0.0	1	0.3	0	0.0	1	0.1
Pregnancy	8	2.7	7	1.9	18	6.3	33	3.5
HIV infection	2	0.7	0	0.0	1	0.3	3	0.3
Hepatitis C antibody positive	0	0.0	2	0.6	1	0.3	3	0.3
Other	0	0.0	3	0.8	0	0.0	3	0.3

Percentages shown as a proportion of either total screened, total analyzed at visit 1, or total that attended visit 2.

# Volunteers may have multiple screen-out reasons therefore columns may sum to >100%.

**Figure 1:**
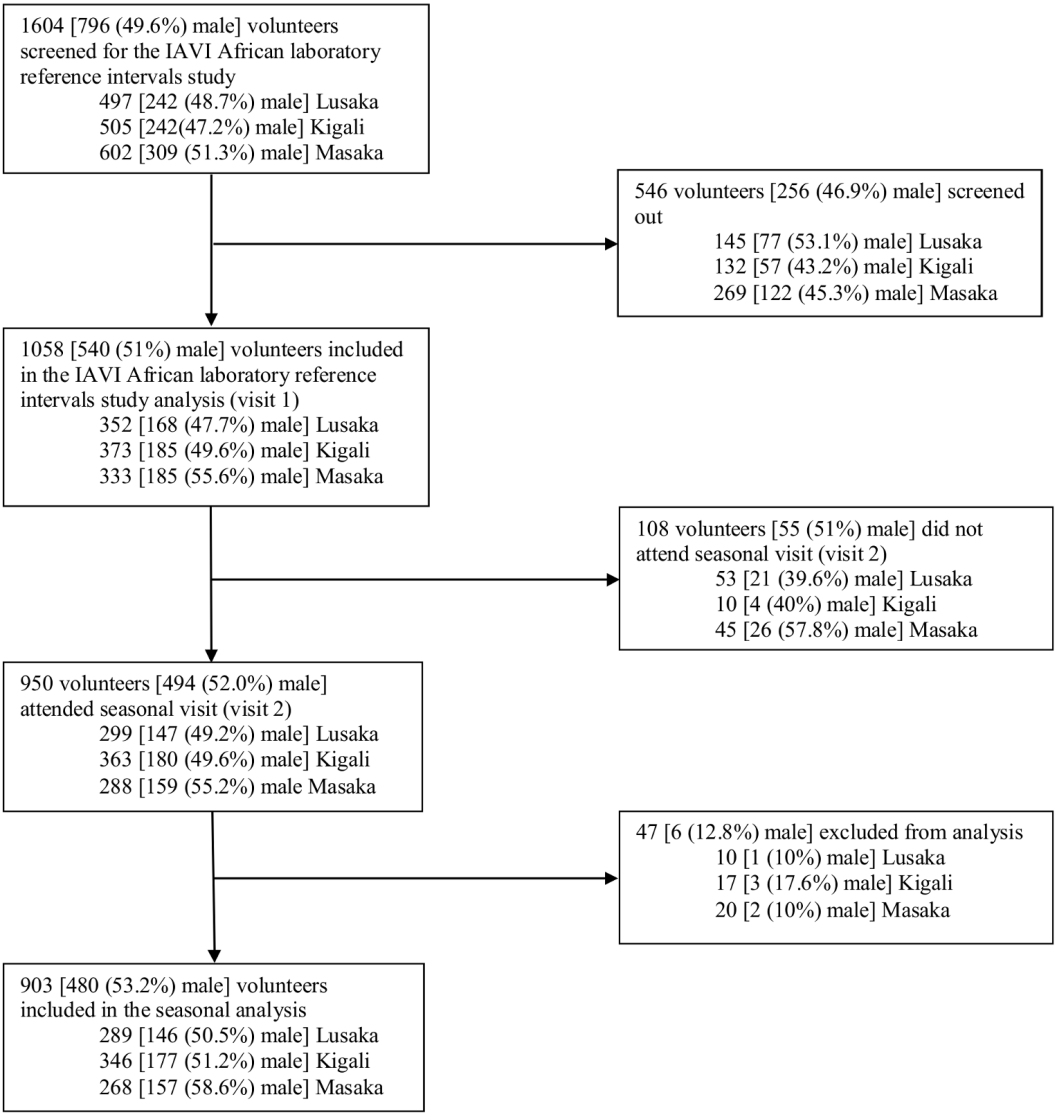
Study Profile.

Of those that were included in the reference intervals analysis (visit 1), 950 [Lusaka (299), Kigali (373), and Masaka (333)] volunteers returned for the seasonal visit. Reasons for not attending the seasonal visit were loss to follow-up (102), investigator initiated termination following volunteer non-compliance to study requirements (2) and volunteer request to discontinue/refusal (4). Of those that attended the seasonal visit, 47 [Lusaka (10), Kigali (17), and Masaka (20)] volunteers were excluded from analysis due to: pregnancy (33), clinically significant physical examination findings (4), HIV infection (3), presence of hepatitis C antibodies (3), hospitalization in past 6 months (1) and other reasons (3) (Table 1). Thus, 903 [Lusaka (289), Kigali (345), and Masaka (268)] volunteers were eligible for inclusion in the current analysis (Figure 1).

Of those included in the analysis, 480 (53.2%) were male, 564 (62.4%) lived in an urban setting, 227 (25.2%) had secondary or higher education, 525 (58.1%) were subsistence farmers, 109 (12.1%) were smokers, and 328 (36.4%) reported alcohol consumption (Table 2).

**Table 2:** Volunteer baseline characteristics.

Characteristic	Sub category	visit 1‡		visit 2†	
		N	%	N	%
Sex	Male	538	50.8	480	53.2
	Female	520	49.2	423	46.8
Age (years)	17–24	302	28.5	238	26.4
	25–34	464	43.9	405	44.8
	35+	292	27.6	260	28.8
Site	Kigali	373	35.3	346	38.3
	Masaka	333	31.5	268	29.7
	Lusaka	352	33.2	289	32.0
Environment	Urban	643	60.8	564	62.4
	Semi Urban	117	11.1	101	11.2
	Rural	298	28.1	238	26.4
Education	None	115	10.8	97	10.7
	Incomplete primary	318	30.1	279	30.9
	Primary	348	32.8	300	33.2
	High/Secondary	222	21.0	181	20.1
	>High/Secondary	55	5.2	46	5.1
Occupation	Subsistence farmer	603	57.0	525	58.1
	Petty trader	38	3.6	31	3.4
	Unskilled labour	312	29.5	256	28.4
	House wife	84	7.9	72	8.0
	Professional	12	1.1	10	1.1
	Other	9	0.8	9	1.0
Smoking status	Smoker	128	12.1	109	12.1
	Non-smoker	930	87.9	794	87.9
Alcohol consumption	Yes	373	35.3	328	36.4
	No	685	64.7	575	63.6

‡ Volunteers included in the IAVI African laboratory reference intervals study analysis.

† Volunteers that attended the seasonal visit and were included in the seasonal analysis study.

### Applying consensus rainy season reference intervals to dry season values

#### Haematology

The proportion of volunteers whose dry season haematology values were out-of-range (OOR) when applied to the consensus rainy season reference intervals ranged from 2.7% (platelets) to 8.1% (RBC counts, men) (Table 3).

**Table 3:** Consensus seasonal haematology parameter reference intervals and out-of-range values.

		Rainy season		Dry season	
Parameter	N	Consensusreferenceinterval	OOR dry‡ N (%)	Consensusreferenceinterval	OOR rainy‡‡ N (%)
Hemoglobin, g/dl					
Male	480	12.3–17.4	19 (3.9)	12.3–17.6	24 (5.0)
Female	423	10.3–15.4	23 (5.4)	10.5–15.6	19 (4.5)
Hematocrit, %					
Male	480	36.0–50.3	22 (4.6)	36.3–51.4	30 (6.3)
Female	423	30.8–45.2	22 (5.2)	31.7–45.5	19 (4.5)
RBC, 10^6^ cells/µL					
Male	480	3.9–6.1	39 (8.1)	4.1–6.1	17 (3.5)
Female	423	3.7–5.6	20 (4.7)	3.8–5.7	18 (4.3)
Platelets, 10^3^ cells/µL	903	132.5–396.8	24 (2.7)	107.8–419.0	76 (8.4)
MCV, fl	903	71–100	42 (4.7)	70–98	34 (3.8)
WBC, 10^3^ cells/µL	903	3.2–8.1	42 (4.7)	3.1–8.2	49 (5.4)
Neutrophils, 10^3^ cells/µL	903	1.0–4.4	35 (3.9)	1.0–4.6	44 (4.9)
Lymphocytes, 10^3^ cells/µL	903	1.2–3.6	53 (5.9)	1.2–3.5	39 (4.3)
Monocytes, 10^3^ cells/µL	903	0.20–0.74	46 (5.1)	0.19–0.70	38 (4.2)
Eosinophils, 10^3^ cells/µL*	634	0.04–1.18	30 (4.7)	0.03–1.18	62 (9.8)
Basophils, 10^3^ cells/µL∧	613	0.01–0.11	30 (4.9)	0.01–0.12	34 (5.5)
CD4 T cells, cells/µL	903	469.2–1595.6	59 (6.5)	464.1–1472.7	34 (3.8)
CD8 T cells, cells/µL	903	241.4–1051.6	49 (5.4)	239.4–1059.6	47 (5.2)

‡ The number and percentage of volunteers whose dry season values are outside the consensus rainy season reference interval.

‡‡ The number and proportion of volunteers whose rainy season values are outside the consensus dry season reference interval.

*Excludes all Masaka volunteers (Masaka eosinophil counts varied significantly from those of other sites) and one Kigali volunteer with a missing value.

∧ Excludes all Lusaka volunteers (Lusaka basophil counts varied significantly from those of other sites) and one volunteer with a missing value at Kigali.

#### Biochemistry

The proportion of volunteers whose dry season biochemistry values were OOR when applied to the consensus rainy season reference intervals ranged from 2.8% (LDH) to 7.0% (albumin) (Table 4).

**Table 4:** Consensus seasonal biochemistry parameter reference intervals and out-of-range values.

Parameter	N	Rainy season		Dry season	
		Consensus referenceinterval	OORdry‡N (%)	Consensus referenceinterval	OORrainy‡‡N (%)
AST, IU/l	903	14–54	51 (5.7)	13.4–51.0	43 (4.8)
ALT, IU/l	903	10.0–54.7	35 (3.9)	8.0–48.8	49 (5.4)
ALP, IU/l*	634	48.2–169.6	41 (6.5)	48.9–156.2	24 (3.9)
Direct bilirubin, µmol/L	903	0.7–9.6	47 (5.2)	0.7–9.6	50 (5.5)
Total bilirubin, µmol/L	903	2.8–35.2	39 (4.3)	2.7–34.2	43 (4.8)
Albumin, g/L	903	36–51	64 (7.0)	34–50	70 (7.8)
Total lgG, mg/dL*	634	1098.5–2454.3	25 (3.9)	1067.8–2617.5	55 (8.7)
Total protein, g/L	903	51.5–84	62 (6.9)	59–85	34 (3.8)
Creatinine, µmol/L	903	45.5–110.0	35 (3.9)	46.4–117.3	56 (6.2)
Total amylase, IU/L	903	36–160	26 (2.9)	33.5–171.0	58 (6.4)
CPK, IU/L					
Male	480	67.8–554.2	30 (6.3)	69.3–555.8	29 (6.0)
Female	423	53.9–350.6	25 (5.9)	59–396	22 (5.2)
LDH, IU/L*	634	233.1–562.7	18 (2.8)	123.0–559.2	141 (22.2)

‡ The number and proportion of volunteers whose dry season values are outside the consensus rainy season reference interval.

‡‡ The number and proportion of volunteers whose rainy season values are outside the consensus dry season reference interval.

*Excludes all Masaka volunteers (Masaka ALP, total IgG and LDH values varied significantly from those of other sites) and one volunteer with a missing value at Kigali.

### Applying consensus dry season reference intervals to rainy season values

#### Haematology

The proportion of volunteers whose rainy season haematology values were OOR when applied to the consensus rainy season reference intervals ranged from 3.5% (RBC counts, men) to 9.8% (Eosinophils) (Table 3).

#### Biochemistry

Except for LDH, the proportion of rainy season biochemistry values that were OOR when applied to dry season reference intervals ranged from 3.8% (total protein) to 8.7% (total IgG) (Table 4). About 22% of rainy season LDH values were OOR when applied to the dry season reference interval. Rainy season LDH values were higher than dry season values i.e. 95% lower bound of 233.1 IU/L (rainy season) compared to 123 IU/L (dry season) and upper bound of 562.7 IU/L (rainy season) compared to 559.2 IU/L (dry season).

### Comparison of rainy and dry season haematology and biochemistry parameter means

#### Haematology

The largest difference between consensus rainy and dry season mean haematology values was observed for eosinophils (Table 5). The percentage difference between consensus seasonal mean eosinophil counts was 40.0% with the higher value observed in the rainy season. In the site stratified analysis (Table 6), mean eosinophil counts were higher in the rainy compared to the dry season at Kigali and Lusaka with percentage differences of 9.5% and 5.4% respectively. At Masaka however, mean eosinophil count was higher in the dry compared to the rainy season (percentage difference: 2.6%). Percentage differences between consensus seasonal mean values for other WBC subsets ranged from 0.0% (neutrophils, monocytes, basophils) to 4.7% (lymphocytes) (Table 5). Within sites however, larger but inconsistent seasonal variations were observed for some of the parameters (Table 6). For example, mean basophil counts did not vary by season at Lusaka and Masaka but were higher in the dry compared to the rainy season at Kigali (percentage difference: 22.2%). Mean monocyte counts were higher in the rainy compared to the dry season at Kigali and Lusaka with percentage differences of 4.9% and 8.5% respectively. In contrast, mean monocyte count was higher in the dry compared to the rainy season at Masaka (percentage difference: 11.5%). Mean lymphocyte counts were higher in the rainy compared to the dry season at Lusaka but did not change at Kigali and Masaka. At all sites, mean CD4 (except Lusaka) and CD8 T cell counts were higher in the rainy compared to the dry season. The largest percentage differences were observed at Kigali [CD4 (4.8%); CD8 (6.1%)]. The largest variation between seasonal platelet counts was observed at Lusaka (percentage difference: 5.5%) with higher values observed in the dry season. In contrast, platelet counts were higher in the rainy compared to the dry season at Kigali and Masaka with percentage differences of 3.5% and 0.8% respectively.

**Table 5:** Comparison of consensus rainy and dry season haematology parameter mean values.

		Rainy season	Dry season	
Parameter	N	Mean, median(min–max)	Mean, median(min–max)	Percentage differencebetween seasonalmeans
Hemoglobin, g/dl				
Male	480	15, 15.2 (10.7–18.4)	15.2, 15.3 (9.7–18.9)	1.3
Female	423	13.3, 13.4 (8.3–16.3)	13.4, 13.6 (7.8–16.4)	0.7
Hematocrit, %				
Male	480	44.1, 44.2 (30.6–52.6)	44.6, 44.8 (28.5–56.2)	1.1
Female	423	39.1, 39.4 (26.7–48.5)	39.6, 40.1 (26.0–47.7)	1.3
RBC, 10^6^ cells/µL				
Male	480	5.0, 5.0 (3.5–6.7)	5.1, 5.1 (3.2–6.9)	2.0
Female	423	4.6, 4.6 (3.4–6.1)	4.7, 4.7 (3.1–6.1)	2.2
Platelets, 10^3^ cells/µL	903	242.8, 235.0 (103.0–563.0)	244.2, 238.0 (102.0–572.0)	0.6
MCV, fl	903	87.1, 88.0 (61.0–110.0)	86.6, 87.0 (60.1–109.0)	0.6
WBC, 10^3^ cells/µL	903	5.2, 5.1 (2.3–9.6)	5.2, 5.0 (1.9–9.9)	0.0
Neutrophils, 10^3^ cells/µL	903	2.3, 2.1 (0.7–5.9)	2.3, 2.1 (0.7–6.0)	0.0
Lymphocytes, 10^3^ cells/µL	903	2.2, 2.1 (0.8–4.8)	2.1, 2.0 (0.8–6.7)	4.7
Monocytes, 10^3^ cells/µL	903	0.4, 0.4, (0.1–2.2)	0.4, 0.4 (0.1–1.5)	0.0
Eosinophils, 10^3^ cells/µL*	634	0.3, 0.2 (0.02–2.3)	0.2, 0.2 (0.0–2.1)	40.0
Basophils, 10^3^ cells/µL∧	613	0.04, 0.04 (0.01–0.30)	0.04, 0.04 (0.01–0.50)	0.0
CD4 T cells, cells/µL	903	912.5, 872.5 (269.0–1798.0)	892.1, 865.0 (149.0–1762.0)	2.3
CD8 T cells, cells/µL	903	563, 526 (150–1285)	542.9, 512.0 (158.0–1278.0)	3.6

*Excludes all Masaka volunteers (Masaka eosinophil counts varied significantly from those of other sites) and one volunteer with a missing value at Kigali.

∧ Excludes all Lusaka volunteers (Lusaka basophil counts varied significantly from those of other sites) and one volunteer with a missing value at Kigali.

**Table 6:** Comparison of site rainy and dry season haematology parameter mean values.

	Kigali				Masaka				Lusaka			
Parameter	N	Rainyseasonmean	DrySeasonmean	Percentagedifference	N	Rainyseasonmean	Dryseasonmean	Percentagedifference	N	Rainyseasonmean	DrySeasonmean	Percentagedifference
Hemoglobin, g/dl												
Male	178	15.8	15.6	1.3	155	14.6	14.7	0.7	145	14.6	15.4	5.3
Female	168	13.9	13.7	1.4	113	13.0	12.8	1.6	144	13.0	13.6	4.5
Hematocrit, %												
Male	178	45.8	45.2	1.3	155	42.7	43.1	0.9	145	43.4	45.3	4.3
Female	168	40.4	40.1	0.7	113	38.0	37.6	0.4	144	38.9	40.5	4.0
RBC, 10^6^ cells/µL												
Male	178	5.2	5.1	1.9	155	5.0	5.0	0.0	145	4.8	5.2	8.0
Female	168	4.6	4.5	2.2	113	4.6	4.5	2.2	144	4.6	4.9	6.3
Platelets, 10^3^ cells/µL	346	244.3	235.9	3.5	268	218.9	217.1	0.8	289	243.7	257.4	5.5
MCV, fl	346	88.1	88.4	0.3	268	84.7	85.0	0.4	289	88.3	85.6	3.1
WBC, 10^3^ cells/µL	346	5.0	4.9	2.0	268	5.3	5.4	1.9	289	5.3	5.2	1.9
Neutrophils, 10^3^ cells/µL	346	2.2	2.2	0.0	268	2.0	2.1	4.9	289	2.5	2.6	3.9
Lymphocytes, 10^3^ cells/µL	346	2.1	2.1	0.0	268	2.2	2.2	0.0	289	2.1	2.0	4.9
Monocytes, 10^3^ cells/µL	346	0.42	0.40	4.9	268	0.41	0.46	11.5	289	0.37	0.34	8.5
Eosinophils, 10^3^ cells/µL*	345	0.22	0.20	9.5	268	0.38	0.39	2.6	289	0.19	0.18	5.4
Basophils, 10^3^ cells/µL*	345	0.04	0.05	22.2	268	0.04	0.04	0.0	289	0.02	0.02	0.0
CD4 T cells, cells/µL	346	954	909	4.8	268	914	898	1.8	289	851	853	0.2
CD8 T cells, cells/µL	346	595	560	6.1	268	528	527	0.2	289	547	534	2.4

*One missing value at Kigali.

Percentage differences between consensus rainy and dry season mean haemoglobin and RBC count values ranged from 0.7% (haemoglobin, men) to 2.2% (RBC, women). However, seasonal differences for these parameters were larger at Lusaka [haemoglobin: 5.3% (men); 4.5% (women) and RBC count: 8.0% (men); 6.3% (women)] with lower values observed in the dry season.

#### Biochemistry

The largest differences between consensus rainy and dry season mean values were observed for LDH, CPK (men and women), total amylase, and ALT (Table 7).

**Table 7:** Comparison of consensus rainy and dry season biochemistry parameter mean values.

		Rainy season	Dry season	
Parameter	N	Mean, median(min–max)	Mean, median(min–max)	Percentage differencebetween seasonal means
AST, IU/l	903	25.8, 24.0 (6.0–95.0)	26.4, 24.0 (10.0–90.0)	2.3
ALT, IU/l	903	23.3, 21.0 (7.0–79.0)	21.7, 19.0 (3.0–78.0)	7.1
ALP, IU/l*	634	88.1, 82.0 (36.0–228.0)	89.4, 85.0 (19.0–218.0)	1.5
Direct bilirubin, µmol/L	903	3.5, 2.9 (0.1–13.5)	3.5, 3.0 (0.1–14.6)	0.0
Total bilirubin, µmol/L	903	11.6, 9.2 (0.2–84.8)	12.0, 10.0 (0.1–71.0)	3.4
Albumin, g/L	903	42.7, 42.0 (28.0–89.0)	41.8, 42.0 (23.0–57.0)	2.1
Total lgG, mg/dL*	634	1637.2, 1594.0 (618.0–3247.0)	1645.1, 1591.5 (632.0–4021.0)	0.5
Total protein, g/L	903	69.7, 71.0 (10.0–98.0)	70.0, 70.0 (10.0–98.0)	0.4
Creatinine, µmol/L	903	74.6, 73.0 (24.0–135.0)	75.0, 73.0 (26.0–147.0)	0.5
Total amylase, IU/L	903	78.2, 72.0 (23.0–252.0)	83.8, 77.0 (17.0–323.0)	6.9
CPK, IU/L				
Male	480	195.9, 159.0 (16.0–911.0)	213.2, 172.0 (21.0–938.0)	8.5
Female	423	142.1, 122.0 (40.0–684.0)	151.9, 127.0 (23.0–752.0)	6.7
LDH, IU/L*	634	347.6, 331.5 (186.0–947.0)	315.2, 318.5 (81.0–763.0)	9.8

*Excludes all Masaka volunteers (Masaka ALP, total IgG and LDH values varied significantly from those of other sites) and one volunteer with a missing value at Kigali.

The percentage difference between consensus seasonal mean LDH concentrations was 9.8% with the higher value observed in the rainy season. At the site level (Table 8); mean LDH values were higher in the rainy compared to the dry season at Kigali and Lusaka with percentage differences of 2.5% and 21.7% respectively. Conversely, mean LDH concentration was higher in the dry compared to the rainy season at Masaka (percentage difference: 3.3%).

**Table 8:** Comparison of site rainy and dry season biochemistry parameter mean values.

	Kigali				Masaka				Lusaka			
Parameter	N	Rainyseasonmean	DrySeasonmean	Percentagedifference	N	Rainyseasonmean	Dryseasonmean	Percentagedifference	N	Rainyseason mean	Dryseasonmean	Percentagedifference
AST, IU/l	346	25.2	25.3	0.4	268	28.0	28.8	2.8	289	24.9	25.3	1.6
ALT, IU/l	346	22.7	21.2	6.8	268	22.9	23.5	0.0	289	24.3	20.2	18.4
ALP, IU/l*	345	86.3	90.5	4.8	268	204.9	209.0	2.0	289	94.3	92.7	1.7
Bilirubin direct, µmol/L	346	3.2	3.7	14.5	268	4.2	4.0	4.9	289	3.3	2.9	12.9
Bilirubin total, µmol/L	346	13.1	13.6	3.7	268	12.9	13.1	1.5	289	8.1	9.1	11.6
Albumin, g/L	346	41.9	39.4	6.2	268	42.6	41.7	2.1	289	43.5	45.0	3.4
Total lgG, mg/dL*	345	1669	1675	0.4	268	2145	2164	0.9	289	1600	1609	0.6
Total protein, g/L	346	70.5	71.3	1.1	268	69.2	69.3	0.1	289	70.3	70.1	0.3
Creatinine, µmol/L	346	68.2	70.2	2.9	268	78.8	76.3	3.2	289	80.0	80.0	0.0
Amylase, IU/L	346	78.0	79.0	1.3	268	90.0	91.0	1.1	289	70.0	84.0	18.2
CPK												
Male	178	173	208	18.4	155	216	196	9.7	145	196	237	18.9
Female	168	131	146	10.8	113	157	144	8.6	144	145	171	16.5
LDH*	345	364	355	2.5	268	481	497	3.3	289	332	267	21.7

*One missing value at Kigali.

Percentage differences between consensus seasonal mean CPK concentrations were 8.5% (men) and 6.7% (women) with the higher values observed in the dry season. Mean CPK values were higher in the dry compared to the rainy season at Lusaka [percentage difference: 18.9% (men); 16.5% (women)] and Kigali [percentage difference: 18.4% (men); 10.8% (women)]. In contrast, mean CPK values were higher in the rainy compared to the dry season at Masaka [percentage difference: 9.7% (men); 8.6% (women)].

The percentage difference between consensus seasonal mean total amylase concentrations was 6.9% with the higher value observed in the dry season. Site mean total amylase concentrations were also higher in the dry compared to the rainy season [percentage difference: 1.3% (Kigali); 18.2% (Lusaka); 1.1% (Masaka)].

The percentage difference between consensus seasonal mean ALT concentrations was 7.1% with the higher value observed in the rainy season. Mean ALT values were higher in the rainy compared to the dry season at Kigali and Lusaka with percentage differences of 6.8% and 18.4% respectively. Rainy and dry season mean ALT values were similar at Masaka.

Percentage differences between consensus seasonal mean values for other biochemistry parameters ranged from 0.0% (direct bilirubin) to 3.4% (total bilirubin). However, larger seasonal variations were observed for some of these biochemistry parameters at the site level (Table 8). Mean direct bilirubin concentrations were higher in the rainy compared to the dry season at Lusaka and Masaka with percentage differences of 12.9% and 4.9% respectively. Conversely, mean direct bilirubin concentration was higher in the dry compared to the rainy season at Kigali (percentage difference: 14.5%). Mean total bilirubin concentrations were higher in the dry compared to the rainy season at all sites but the largest variation was observed at Lusaka (percentage difference: 11.6%). Mean albumin values were higher in the rainy compared to the dry season at Kigali and Masaka with the largest variation observed at Kigali (percentage difference: 6.2%). In contrast, mean albumin level was higher in the dry compared to the rainy season at Lusaka (percentage difference: 3.4%).

## Discussion

The main finding of this study is that seasonal variation may not significantly impact values of clinical laboratory parameters among healthy adults in Rwanda, Zambia, and Uganda. We found that for more than 90% of volunteers, haematology and biochemistry parameter values obtained in the dry season were within the consensus rainy season reference interval. With the exception of LDH, values obtained in the rainy season were also within the consensus dry season reference interval for more than 90% of volunteers. Furthermore, differences between rainy and dry season mean haematology and biochemistry parameter values were modest and unlikely to have clinical significance.

Mean total WBC counts did not vary by season at the consensus level and only varied by about 2% within sites. Among WBC subset counts however, larger seasonal variations were observed for eosinophils, basophils, monocytes, and CD8 T lymphoctyes. The magnitude and direction of variation were however inconsistent across sites. For example, seasonal eosinophil counts varied by more than 5% at Kigali and Lusaka with higher counts recorded in the rainy season but an opposite and smaller effect was observed at Masaka. Basophil counts varied by 22% between seasons at Kigali but were unaffected by season at Masaka and Lusaka. Seasonal variation in WBC subset counts has been reported in other populations [4], [16], [17], [24], and may be due to a variety of factors including; seasonal variation in the burden of endemic infections [17], effects of neurohormones and adrenocorticosteroids [8], [25], changes in iron binding proteins [8], menstrual cycle [8], and environment conditions such as stress [4], [8], exposure to cold and sunlight [8], and seasonal allergen exposure [24].

Variability between seasonal platelet counts was generally small at all the sites. At Kigali and Masaka, platelet counts were higher in the rainy compared to the dry season contrary to the effect observed at Lusaka. Seasonal variation in platelet counts has been reported among healthy adults in other settings. In Italy, higher counts were reported in the winter-autumn compared to spring-summer period [26]. In contrast, higher counts were reported in the summer compared to other seasons in China [27].

About 22% of rainy season LDH values were outside the consensus dry season reference interval. Mean LDH values were higher in the rainy compared to dry season at Kigali and Lusaka but the opposite effect was observed at Masaka. The percentage differences between seasonal mean LDH values for Kigali and Masaka were small however at approximately 3% compared to 22% for Lusaka. LDH is significantly elevated in diseases affecting erythrocytes, liver, heart, skeletal muscles and kidneys [28]. The increase in LDH levels observed in the rainy season at Lusaka may be due to hepatocellular injury and red cell haemolysis associated with increased malaria infections in this season. The relatively low RBC and high direct bilirubin levels observed in the rainy season at Lusaka probably signify increased haemolyis in this season. Although malaria is endemic in all the study populations with year round transmission, peak incidence of clinical malaria occurs during the rainy season [29]–[31]. We however did not investigate malaria parasitaemia in this study and therefore cannot confirm these associations. These findings suggest that HIV biomedical prevention and other clinical trials in malaria endemic countries should include tests for malaria parasitaemia as this may affect some outcomes.

Differences greater than 5% between seasonal mean CPK values were observed for men and women at all the study sites. Except for Masaka, higher values were observed in the dry compared to rainy season. Seasonal variation of CPK values has been observed in other settings [32], [33]. The reason for this seasonal effect is not well understood, but may be related to the degree of physical activity or hormonal regulation [33]. The magnitude of the seasonal effect was bigger for men compared to women at each study site. The attenuated increase of CPK in women may be partly explained by the effect of circulating estrogens on skeletal muscle [33]–[35] although the mechanisms are poorly understood [35].

Mean total amylase concentrations were higher in the dry compared to the rainy season at all study sites. However, the magnitude of variation was quite small at Kigali and Masaka compared to Lusaka. Evidence from studies among healthy adults shows that levels of salivary α-amylase may be increased in response to psychological and physical stress including exercise, heat and cold [36]. We however did not differentiate between pancreatic and salivary amylase forms and are therefore unable to attribute the increase in the total serum amylase concentration observed in our study to either or both forms.

Except for total and direct bilirubin, and ALT, seasonal variation among liver function tests was minimal. Mean total bilirubin concentrations were higher in the dry compared to the rainy season at all sites although the variation observed at Kigali and Masaka was small. Direct bilirubin concentrations were higher in the rainy compared to the dry season at Lusaka and Masaka but contrary results were obtained at Kigali. Total bilirubin has been reported to increase in the summer in other settings [37]. Mean ALT concentrations were higher in the rainy compared to the dry season at Kigali and Lusaka but remained unchanged at Masaka. Seasonal variation of ALT may be attributed to seasonal changes in vascular tone, climatic stress, hormones, and alcohol intake [37].

The inter-site discrepancies observed in the direction and degree of seasonal impact for some of the laboratory parameters may be partly due to geographical, topographical, climatic, and population differences. These differences may influence the incidence and intensity of infections, exposure to cold, sunlight and allergens, levels of physical activity, diet, lifestyle, and other factors associated with seasonal variation of laboratory parameter values. Of note, Lusaka which is farther from the equator (latitude 15.4° south) compared to Masaka (latitude 0.3° south) and Kigali (latitude 1.9° south) tended to have the largest seasonal impact on laboratory parameter values. In general, seasonal variation in exposure to ultraviolet radiation increases with distance from the equator [38], [39]. Therefore, it is probable that changes in laboratory parameter values that are to some extent dependent on the level of exposure to ultraviolet radiation would be more pronounced in populations living at higher latitudes compared to those located close to the equator.

Our study had some limitations that may have affected the extent to which seasonal impact on laboratory parameters could be evaluated. As mentioned above, seasons were pre-defined by site investigators based on their knowledge of the local seasonal patterns and existing literature. Additionally, rain gauge data collected during the study were used to retrospectively confirm seasonal assignments (Lusaka and Masaka). Nevertheless, the possibility of inaccurate or inconsistent seasonal assignments exists since we did not use standardised criteria for definition of seasons. Also, sample collection did not take into account the possibility that seasonal impact on laboratory parameter values may not manifest until several weeks or months into a given season. For example the maximum haemolytic effect of malaria would be expected to coincide with the peak incidence of clinical malaria which occurs a few weeks following the peak of the rains [29], [30].

In summary, we found that among healthy adults in Rwanda, Zambia and Uganda, seasonality had a limited impact on haematology and biochemistry parameters. Seasonal variation may not be an important factor in the evaluation of clinical laboratory parameters in HIV biomedical prevention and other clinical trials in these countries.
